# Compassionate Conservation is indistinguishable from traditional forms of conservation in practice

**DOI:** 10.3389/fpsyg.2022.750313

**Published:** 2022-10-03

**Authors:** Christopher A. Bobier, Benjamin L. Allen

**Affiliations:** ^1^Department of Theology and Philosophy, Saint Mary's University of Minnesota, Winona, MN, United States; ^2^Institute for Life Sciences and the Environment, University of Southern Queensland, Toowoomba, QLD, Australia; ^3^Centre for African Conservation Ecology, Nelson Mandela University, Port Elizabeth, South Africa

**Keywords:** lethal control, wildlife management, decision-making, animal welfare, virtue ethics, invasive species

## Abstract

Animal welfare and ethics are important factors influencing wildlife conservation practice, and critics are increasingly challenging the underlying ethics and motivations supporting common conservation practices. “Compassionate Conservationists” argue that all conservationists should respect the rights of individual sentient animals and approach conservation problems from a position of compassion, and that doing so requires implementing practices that avoid direct harm to individual animals. In this way Compassionate Conservationists seek to contrast themselves with “Traditional Conservationists” who often express consequentialist decision-making processes that ostensibly aim to dispassionately minimize net animal harms, resulting in the common use of practices that directly harm or kill some animals. Conservationists and other observers might therefore conclude that the two sides of this debate are distinct and/or that their policy proscriptions produce different welfare outcomes for animals. To explore the validity of this conclusion we review the ethical philosophies underpinning two types of Compassionate Conservation—deontology and virtue ethics. Deontology focusses on animal rights or the moral duties or obligations of conservationists, whereas virtue ethics focusses on acting in ways that are virtuous or compassionate. We demonstrate that both types permit the intentional harm and killing of animals when faced with common conservation problems where animals will be harmed no matter what the conservationist does or does not do. We then describe the applied decision-making processes exhibited by Compassionate Conservationists (of both types) and Traditional Conservationists to show that they may each lead to the implementation of similar conservation practices (including lethal control) and produce similar outcomes for animals, despite the perceived differences in their ethical motivations. The widespread presence of wildlife conservation problems that cannot be resolved without causing at least some harm to some animals means that conservationists of all persuasions must routinely make trade-offs between the welfare of some animals over others. Compassionate Conservationists do this from an explicit position of animal rights and/or compassion, whereas Traditional Conservationists respect animal rights and exhibit this same compassion implicitly. These observations lead to the conclusion that Compassionate Conservation is indistinguishable from traditional forms of conservation in practice, and that the apparent disagreement among conservationists primarily concerns the effectiveness of various wildlife management practices at minimizing animal harm, and not the underlying ethics, motivations or morality of those practices.

## Introduction

The conservation and wellbeing of animals is of great concern to many people around the world. People employ a variety of approaches to manage those problems with the general goal of improving the welfare and conservation status of animals, and such efforts to conserve animals have a long history and tradition in many cultures and countries. Recently, Compassionate Conservation has arisen and tried to position itself as an enlightened approach distinct from traditional forms of conservation. Compassionate Conservationists contrast themselves with Traditional Conservationists who ostensibly express consequentialist or utilitarian decision-making processes that seek to dispassionately maximize animal welfare or some other biodiversity conservation value. According to such Traditional Conservationists, who typically value all forms of biodiversity including but not limited to plants, invertebrates *and* sentient animals, it is not especially concerning to employ lethal or harmful conservation practices when doing so clearly minimizes harm or maximizes overall animal welfare outcomes (see Dubois et al., 2017; Driscoll and Watson, 2019; Hampton et al., 2019; Johnson et al., 2019; Oommen et al., 2019). For example, killing a relatively small number of invasive predators to avert the killing or loss of relatively large numbers of prey shows no favoritism toward a particular species and demonstrably maximizes overall animal welfare; it is therefore a common approach used in many traditional forms of animal conservation (e.g., Russell et al., 2016; Allen and Hampton, 2020). Compassionate Conservationists, by contrast, argue for the adoption of what they claim is a more compassionate point of view (see Vucetich and Nelson, 2007; Ramp, 2013; Ramp et al., 2013; Ramp and Bekoff, 2015; Wallach et al., 2015, 2018, 2020a,b; Bekoff, 2017, 2020; Bekoff and Pierce, 2017; Ben-Ami, 2017; University of Technology Sydney, 2019; Batavia et al., 2020, 2021; Nelson et al., 2021). Rather than approach conservation from a dispassionate lens of maximizing desirable consequences for collectives such as populations, species or biodiverse ecosystems, Compassionate Conservationists instead argue that we should approach conservation issues from a position of compassion that avoids human involvement in direct harm to individual animals. This is because the proper object of compassion is an individual and it is not compassionate to another individual to deliberately harm or kill them. Such a view therefore encourages practices that allow invasive predators to freely kill and cause extinctions of their prey because it would not be compassionate for humans to kill the predators (e.g., Wallach et al., 2015; Ben-Ami, 2017). Conservationists of all types may value animal welfare and biodiversity conservation, but the picture that emerges is that Compassionate Conservationists rarely, if ever, support the adoption of lethal or harm-causing policies while Traditional Conservations often do.

Despite this apparent conflict between the two approaches, here we argue that the manifest decision-making processes of a Compassionate Conservationist are indistinct from a Traditional Conservationist in practice, and that both approaches yield similar outcomes for animals. With a focus on sentient animals to show this, we first distinguish two versions of the ethical foundation of Compassionate Conservation—deontological and virtue-based—and show that each version allows for the adoption of conservation policies that involve intentional animal harm. This indicates that Compassionate Conservationists cannot oppose policies that involve intentional death or animal harm solely on ethical grounds. Having delineated these two ethical foundations, we then argue that, given the non-ideal world we live in where it is often the case that animals will be harmed no matter what policies the conservationist adopts (Bobier and Allen, 2022).^1^ Compassionate Conservationists espousing either version will adopt similar decision-making procedures to Traditional Conservationists: all will seek to minimize animal harm or maximize compassion in a given situation. Although the emphasis of the approaches may differ according to their distinct motivations, their practical outcomes for animals do not vary as much as might be supposed. This begs the question: what is all the fuss about? Why are Compassionate Conservations so insistent that their approach produces better outcomes for animals than traditional forms of conservation practice? Do Traditional Conservationists realize that they have been exhibiting Compassionate Conservation philosophy all along? Our aim is to explore these questions with the hope that the different points of view may be reconciled to some degree.

## What are deontology, virtue ethics, and consequentialism?

Deontology (or Kantian ethics), virtue ethics, and consequentialism are three different ethical theories that describe the different considerations of what makes an action morally right or wrong, or praiseworthy or blameworthy (Sandel, 2010; Shafer-Landau, 2012). Deontologists assess actions based on whether the action conforms to one’s duty (e.g., do not lie), with duties being grounded in and identified by the rights of persons to be respected (e.g., not lied to; Kant, 1785). Animal ethicists inspired by deontology argue that animals have certain rights, which often are or should be the same rights afforded to humans, and that humans should therefore act in ways that respect the rights of animals (Regan, 1983; Cochrane, 2007; Steiner, 2008; Wrenn, 2016). In the context of wildlife conservation, they claim that animals have a right to live free of intentional harm or fear from humans, so humans have a duty to approach conservation in ways that do not intentionally harm animals. Virtue ethicists focus on whether a person’s decisions or actions manifest or are in accordance with virtue, for virtuous living is integral to living a flourishing life (Hursthouse, 1999; Aristotle, 2009; Annas, 2011). Virtue ethicists seek to act in ways that manifest moral character, which is integral to the flourishing of human beings and their natural cosmos in which they are part, and for precisely this reason the appropriate action to exercise of virtues toward others is critical. Compassion, justice, temperance, and so on are important virtues in our treatment of others, so virtue ethicists seek to act in ways that exhibit these virtues toward others. In the context of wildlife conservation, they assert that virtuous people should act virtuously and make appropriate choices that demonstrate compassion toward animals, which typically involves an active refrain from killing or otherwise harming them (Hursthouse, 2006, 2011). Consequentialism does not focus on rights or duties like deontology or on character traits like virtue ethics, but focuses instead on the outcomes or consequences of an action or inaction: an action is good if it maximizes good outcomes and/or minimizes bad outcomes (Mill, 1861; Driver, 2012). A common form of consequentialism posits that the good and bad outcomes are the result of the total amount of pleasure and pain produced, and so animal ethicists wedded to consequentialism attempt to quantify the potential amounts of animal harm occurring in a given context before seeking to act in ways that minimize those harms (Singer, 1993). This means, for example, that harming or killing one animal to save another is acceptable if it avoids more harm than it causes, even if doing so appears to be uncompassionate or a violation of animal rights. Although much more can be said to elaborate on these three theories (see below), it is important to note that Compassionate Conservationists consider themselves to be distinct from Traditional Conservationists—the distinction being that Traditional Conservationists are wedded to consequentialist thinking whereas Compassionate Conservationists are not (Ramp, 2013; Ramp and Bekoff, 2015; Wallach et al., 2015, 2018). Compassionate Conservationists assert that they are not committed to any one ethical theory, consequentialism aside (Beausoleil, 2020; Santiago-Ávila and Lynn, 2020; Coghlan and Cardilini, 2021), and indeed the literature suggests they rely on two ethical foundations: deontology and virtue ethics.

## Deontological-based compassionate conservation

Compassionate Conservationists sometimes appeal to rules or duties, with some advocating for a prohibition on adopting any policies and practices that intentionally harm animals. Bekoff and Ramp (2014) propose that compassion should lead us to adopt a “first-do-no-harm” principle. Wallach et al. (2020a,b) explain that compassion toward animals motivates one to minimize animal suffering, “but not by intentionally harming” them. Bekoff (2017) argues that “killing is not an option” for the Compassionate Conservationist, while Vucetich and Nelson (2007) propose the Golden Rule for conservationists, namely, to treat animals the way the conservationist would want to be treated in the same situation. The basis for this refrain from adopting policies and practices that intentionally harm or kill animals is that non-human animals are also sentient, and sentience matters morally. For instance, Wallach et al. (2020a) defend this view on grounds that sentient animals are persons, and so share the same or similar moral status as human beings; just as the compassionate person would not accept the lethal control of human populations, so also the compassionate person would not accept the lethal control of non-human populations: sentient beings have the right to not be harmed. Accordingly, we should “avoid deliberately harming sentient beings in conservation programs” and “the interests and agency of all sentient beings should be protected in conservation practice” (Wallach et al., 2020a).

Fleming (2018) points out that this type of Compassionate Conservation is simply “animal liberation dressed up as conservation science” and “has little foundation in biology.” Bekoff and Ramp (2014) and Bekoff (2015, 2018) attempt to deny that they are adopting an animal rights position, but this denial is difficult to reconcile with their discussion of a “moral imperative” to foster peaceful coexistence and defense of the “first-do-no-harm” principle on grounds that animals are sentient. It is therefore clear to anyone familiar with the animal ethics literature that this type of Compassionate Conservation bears a striking similarity to the animal rights position (see Cochrane, 2007; Steiner, 2008; Wrenn, 2016). Regan (1983), for example, to whom Wallach et al. (2018) appeal for support, argues that animals that are experiencing-subjects-of-life have intrinsic or inherent value equal to that of human beings, and this creates an obligation on our part to treat animals in ways that respect this inherent value. In other words, animals are subjects of inherent worth and we are obligated to respect their inherent worth; to not do so is morally wrong. Regan argues that this obligation to treat experiencing-subjects-of-life, both humans and non-humans alike, respectfully creates two specific duties. The first is the “do no harm” duty where experiencing-subjects-of-life have a *prima facie* right to not be intentionally or even unintentionally harmed. The second is the “assistance” duty where humans have a responsibility to help experiencing-subjects-of-life that are treated unjustly. Thus, the type of Compassionate Conservation described by Wallach, Bekoff, Ramp, and others clearly appears to be a natural extension of Regan-type deontological, animal rights-based theory. They emphasize that “compassionate conservation incorporates recognition of the intrinsic value of wildlife and the sentience of nonhuman animals” (Bekoff, 2018) and that “individuals are not objects or commodities” that can be sacrificed for the greater good (Bekoff, 2015).

But while deontologists seek to protect individuals from being disrespected or harmed, deontological ethics does allow for cases in which it is permissible to harm an individual for the sake of another. Deontologists assume these are rare or non-ordinary cases, cases in which many sentient beings will be seriously harmed unless the rights of some are violated. Regan (1983) further explains that the right to not be harmed is not an absolute right, and he offers two principles that apply to situations when rights need to be violated. Although Regan believes such situations are rare in ordinary life they are certainly not rare in conservation contexts (see below), and so a deontological ethic may allow the conservationist to regularly violate animal rights in these cases.

The first of Regan’s principles is the “miniride principle.” It posits that we should override the rights of the few in favor of the rights of the many when each individual would be equally harmed. So, for example, in a situation where 51 miners are trapped underground and we can save 50 by killing one, save one by killing 50, or allowing all to die by doing nothing, Regan (1983) defends the permissibility of killing one miner in favor of saving the 50 others. More recently, Abbate (2020) reinforces this view with the principle that “we ought not to treat the basic harm of one as equal to or greater than the basic harms of two or more individuals.” Though the miniride principle might rarely apply in some contexts, deontological-inspired Compassionate Conservationists are commonly faced with the troubling fact that animals will be harmed no matter what conservation policy or practice is adopted (Footnote 1). These are situations in which no matter what the conservationists do or do not do, some animals will be harmed directly and/or indirectly as a result of whatever action or inaction is taken, and Regan’s miniride principle therefore applies in many of these situations (Table 1). For example, if left unmanaged, feral goats (*Capra hircus*) on offshore islands can denude the local vegetation and transform complex forest ecosystems into barren rocky wastelands, causing the local extinction of multiple plant and animal populations, and eventually goats as well (Lee and Stasack, 1993; Márquez et al., 2013; Pafilis et al., 2013). The outcome for all individual animals on the island is starvation and death. Thus, Allen et al. (2021) recently released two dingoes on such an island as a biocontrol tool intended to kill about 300 extant goats in an exercise where the rights of the few (i.e., two dingoes and 300 goats) were overridden by the rights of the many (i.e., the countless individuals from all the other animal species on the island) given that all may have eventually died without intervention.

**Table 1:** Examples of some emblematic conservation problems and how they might be approached from the perspectives of a deontological compassionate conservationist, a virtue-based compassionate conservationist, and consequentialist traditional conservationist.

Example	Deontological compassionate conservation	Virtue-based compassionate conservation	Consequentialist traditional conservation
Killing wolves to protect endangered caribou	Moral desiderata: Would a negative duty to do no harm to wolves trump a positive duty to assist caribou? Will the wolves be harmed if caribou go extinct? Action: Application of the miniride principle may permit the killing of wolves if it is thought that killing a few wolves will prevent the death of wolves and caribou.	Moral desiderata: How can we show compassion to both wolves and caribou? How would the action affect the flourishing of all involved? How would the action affect one’s character? Action: Exercising compassion may permit the killing of wolves depending on the available evidence of the effectiveness of the alternatives.	Moral desiderata: Which approach minimizes the net amount of harm to both wolves and caribou? Action: Killing wolves may be permissible if it minimizes harm.
Shooting overabundant kangaroos to prevent their imminent starvation	Moral desiderata: Would the negative duty to do no harm to kangaroos be overridden by their impending starvation? Action: Application of the worse-off principle may permit killing of some kangaroos to avoid the imminent starvation of more kangaroos.	Moral desiderata: Would leaving all kangaroos to die of starvation be compassionate when it can be prevented by killing some of them? How would the various actions affect the flourishing of all involved? How would the various actions affect one’s character? Action: Killing some kangaroos to alleviate their suffering may be the most compassionate thing to do depending on the available evidence of the effectiveness of the alternatives.	Moral desiderata: Would failure to kill kangaroos before they starve to death result in greater harm to kangaroos? Action: Killing kangaroos may be permissible if it minimizes harm.
Euthanizing a mortally wounded bird	Moral desiderata: Given the negative duty to do no harm to the bird, do any of Regan’s override principles apply? Action: Do not euthanize the wounded bird.	Moral desiderata: Is it compassionate to let the bird suffer longer? How would the various actions affect the flourishing of all involved? How would the various actions affect one’s character? Action: Mercifully euthanizing the bird may be the most compassionate thing to do depending on the available evidence of the effectiveness of the alternatives.	Moral desiderata: Would failure to euthanize the bird cause more harm? Action: Euthanizing the bird would minimize suffering.
Killing invasive rodents to protect seabird nestlings	Moral desiderata: Negative duty to do no harm to rodents, and a positive duty to assist seabirds. Will rodents be harmed if seabirds die? Action: Application of the miniride principle may permit the killing of rodents if it is thought that killing rodents will prevent the death of rodents and seabird nestlings.	Moral desiderata: How can we show compassion to both rodents and seabirds? How would the various actions affect the flourishing of all involved? How would the various actions affect one’s character? Action: Exercising compassion may permit the killing of rodents depending on the available evidence of the effectiveness of the alternatives.	Moral desiderata: Which approach minimizes the net amount of harm to both rodents and seabirds? Action: Killing rodents may be permissible if it minimizes harm.
Killing rabbits with a biocontrol (lethal disease) to protect native fauna from overgrazing and starvation	Moral desiderata: Negative duty to do no harm to rabbits, and a positive duty to assist fauna harmed by competition with rabbits. Will rabbits and fauna be harmed if nothing is done? Action: Application of the miniride principle may permit the killing of rabbits if it is thought that killing rabbits will prevent the starvation of rabbits and fauna.	Moral desiderata: How can we show compassion to both rabbits and other fauna? How would the various actions affect the flourishing of all involved? How would the various actions affect one’s character? Action: Exercising compassion may permit the killing of rabbits depending on the available evidence of the effectiveness of the alternatives.	Moral desiderata: Which approach minimizes the net amount of harm to both rabbits and other fauna? Action: Killing rabbits may be permissible if it minimizes harm.
Releasing dingoes to kill feral goats for the protection of native fauna and prevention of starvation by goats	Moral desiderata: Negative duty to do no harm to dingoes or goats, and a positive duty to assist fauna harmed by goats and alleviate goat suffering. Action: Application of the miniride principle may permit the killing of goats if it is thought that killing feral goats will prevent starvation of fauna and goats.	Moral desiderata: How can we show compassion to dingoes, goats and other fauna? How would the various actions affect the flourishing of all involved? How would the various actions affect one’s character? Action: Exercising compassion may permit the use of dingoes to kill goats depending on the available evidence of the effectiveness of the alternatives.	Moral desiderata: Which approach minimizes the net amount of harm to dingoes, goats and other fauna? Action: Releasing dingoes to kill goats may be permissible if it minimizes harm.
Translocating a population of animals to a new area to avoid extinction in their former area	Moral desiderata: Negative duty to do no harm to the animals, and a positive duty to assist them. Action: Application of the worse-off principle may permit relatively minor harms to the animals to avoid greater harms.	Moral desiderata: Would leaving the animals to go extinct be compassionate when it can prevented by harming a few? How would the various actions affect the flourishing of all involved? How would the various actions affect one’s character? Action: Harming the animals to alleviate their greater suffering may be the most compassionate thing to do depending on the available evidence of the effectiveness of the alternatives.	Moral desiderata: Failure to translocate (harm) the animals will result in greater harms than translocating them. Action: Translocating (harming) animals may be permissible if it minimizes harm.
Birth control, sterilization or neutering overabundant cats and then releasing them	Moral desiderata: Negative duty to do no harm to cats, and a positive duty to assist the animals killed by cats. Action: Application of the worse-off principle may permit relatively minor harms to the cats to avoid greater harms to other animals.	Moral desiderata: Would abstaining from harming cats be compassionate when the deaths of other animals could be prevented by harming a few cats? How would the various actions affect the flourishing of all involved? How would the various actions affect one’s character? Action: Harming cats to alleviate the greater suffering of other animals may be the most compassionate thing to do depending on the available evidence of the effectiveness of the alternatives.	Moral desiderata: Failure to neuter (harm) cats will result in greater harms than neutering them. Action: Neutering (harming) cats may be permissible if it minimizes harm.
Aversive conditioning of predators to protect prey	Moral desiderata: Negative duty to do no harm to predators, and a positive duty to assist their prey. Action: Application of the worse-off principle may permit relatively minor harms to the predators to avoid greater harms to prey.	Moral desiderata: Would abstaining from harming predators be compassionate when the deaths of prey animals could be prevented by harming a few predators? How would the various actions affect the flourishing of all involved? How would the various actions affect one’s character? Action: Harming predators to alleviate the greater suffering of prey animals may be the most compassionate thing to do depending on the available evidence of the effectiveness of the alternatives.	Moral desiderata: Failure to scare or harm predators will result in greater harms to prey. Action: Aversive conditioning of predators may be permissible if it minimizes harm.
Aversive conditioning of elephants to protect the crops of subsistence farmers	Moral desiderata: Negative duty to do no harm to elephants, and a positive duty to assist subsistence farmers. Action: Application of the worse-off principle may permit relatively minor harms to the elephants to avoid greater harms to farmers.	Moral desiderata: Would abstaining from harming elephants be compassionate when the starvation of subsistence farmers could be prevented by harming the elephants? How would the various actions affect the flourishing of all involved? How would the various actions affect one’s character? Action: Harming elephants to alleviate the greater suffering of farmers may be the most compassionate thing to do depending on the available evidence of the effectiveness of the alternatives.	Moral desiderata: Failure to scare or harm elephants will result in greater harms to farmers. Action: Aversive conditioning of elephants may be permissible if it minimizes harm.
Guardian dogs to protect penguins from foxes	Moral desiderata: Negative duty to do no harm to dogs and foxes, and a positive duty to assist penguins. Action: Application of the worse-off principle may permit relatively minor harms to the dogs and foxes to avoid greater harms to penguins.	Moral desiderata: Would abstaining from harming all the animals be compassionate when the deaths of penguins could be prevented by harming dogs and foxes? How would the various actions affect the flourishing of all involved? How would the various actions affect one’s character? Action: Harming dogs and foxes to alleviate the greater suffering of penguins may be the most compassionate thing to do depending on the available evidence of the effectiveness of the available alternatives.	Moral desiderata: Failure to harm dogs and foxes will result in greater harms to penguins. Action: Aversive conditioning of predators may be permissible if it minimizes harm.

Regan’s second principle is the “worse-off principle.” It posits that in situations where either a few animals will be severely harmed or many animals will be only slightly harmed, it is preferable to override the rights of the many. In other words, slight inconvenience to the many is acceptable if it avoids substantial harm to the few. So in a situation in which four humans and a dog are stranded in a lifeboat, and all will die unless one is thrown overboard, Regan (1983) argues that the dog should be thrown overboard because the dog’s death is not a harm comparable to the death of a human. A more mundane example would be that every cow receives a vaccine shot (a slight inconvenience and pain) so that the relative few do not get sick and die in a disease outbreak. Although the worse-off principle might also apply rarely in some contexts, conservationists commonly find themselves in situations where the worse-off principle does apply (Table 1). For instance, the aversive conditioning of all would-be predators and other non-target animals through electric fencing (a slight inconvenience and pain) means that the conservationist may not have to kill the predators or allow them to kill the prey animals protected by the electric fence (Fox and Bekoff, 2011; Ben-Ami and Mjadwesch, 2017; Wallach et al., 2018). Alternatively, male and female animals may be sterilized or given a form of contraception so that lethal control methods targeting just the males do not need to be utilized (Ben-Ami and Mjadwesch, 2017; Villa Branco et al., 2017; see also Table 1).

Compassionate Conservationists are therefore obliged to acknowledge that a deontological, rights-based approach cannot ensure that the conservationist will not adopt policies or practices that intentionally kill or harm animals. Bekoff and Pierce (2017) recognize that “there will be blood. That much is sure.” The principles espoused by this type of Compassionate Conservationist are also consistent with this. For example, the “do no harm” tenet highlights that interventions should be “carefully scrutinized and selectively pursued” (Wallach et al., 2018) and “enjoins conservationists to generally avoid intentionally harming [and] killing sentient animals” (Coghlan and Cardilini, 2021), suggesting that harm-causing policies might indeed be selected by conservationists after careful scrutiny of all the available options. Moreover, the goal of “peaceful coexistence” amounts to a recognition that the conservationist’s first response to conflict should not be to kill but rather “critically examine” one’s practices (Wallach et al., 2018) and then “to reduce potential conflict” (Beausoleil, 2020), which again allows for the possibility that a lethal or harmful policy may be adopted to reduce conflict after careful examination. The “individuals matter” principle further means that the conservationist acknowledges the “intrinsic value of wildlife individuals and resists the tendency to reduce them or their value” to their membership in a collective (Wallach et al., 2018), which is a view still compatible with killing or harming animals in certain situations (Table 1). Hence, the preceding principles, examples, and discussion demonstrate that policies and practices which cause intentional harm to animals, including death, can indeed be compatible with deontological-based Compassionate Conservation (Lynn, 2018; Beausoleil, 2020; Coghlan and Cardilini, 2021).

## Virtue-based compassionate conservation

Proponents of Compassionate Conservation sometimes appeal not to our obligations or duties to sentient animals (see above), but rather to neo-Aristotelian virtue ethics and the virtue of compassion itself (see; Batavia et al., 2020, 2021; Wallach et al., 2020a,b). Virtue ethics is distinct from deontology because the emphasis of virtue ethics is the cultivation of a good character that manifests itself in virtuous action, and not the following of moral rules grounded in moral rights (Aristotle, 2009). The morally right thing to do, according to virtue ethics, is to do whatever the virtuous person would do in the situation (Hursthouse, 1999). The virtues are morally good, praiseworthy character traits acquired through rational training. They are imbedded in the person’s “make up,” so to speak, inclining the person to think, reason, feel, and behave in excellent and praiseworthy ways. The virtue of compassion thus involves a particular affect, proper judgment, and motivation to act (Sandler and Cafaro, 2005; Sandler, 2007; Crisp, 2008; Gilbert, 2017), and it is grounded in our shared relation to others, allowing ourselves to be appropriately affected by the suffering of others. The compassionate person not only recognizes the suffering of another, they also feel the appropriate amount of anguish, distress, or pain at their misfortune; and rather than ignore the suffering of another, the compassionate person is motivated to assist the other in the right kind of way and in the right amount. The compassionate person not only cares about how their own actions affect animals, they also care about harms that they are not directly responsible for or related to (e.g., the treatment of animals in research labs or zoos). Proponents of virtue-based Compassionate Conservation assert that when compassion is cultivated into a virtue and becomes an integral part of conservation practice, it leads the mind to recoil at the suggestion that it might be appropriate for conservationists “to kill or intentionally harm certain kinds of beings in certain ways to meet certain objectives” (Batavia et al., 2021).

As Bobier and Allen (Footnote 1) show, however, the virtue of compassion may still lead a person to adopt intentional lethal or harm-causing conservation policies in many or most conservation contexts (Table 1). While the compassionate person cares a great deal about conservation policies and practices that intend animal harm (e.g., killing predators to protect prey), they also care a great deal about conservation policies and practices that have unintended harms (e.g., harms to prey resulting from refusal to kill predators). The virtuous person respects and cares about all animals as particular individuals, and it is precisely this virtue that can motivate them to adopt conservation policies that advocate for the killing or harming of some of them in certain situations. This includes those in which the virtuously compassionate conservationist must decide which policy to adopt (or not) with full knowledge that animals will be harmed in one way or another no matter what decision is made. Under ideal circumstances, such a person would refrain from adopting a policy that involves them in directly harming animals, but conservation contexts are replete with tragic situations in which animals will be harmed no matter what is done or not done. To be clear, the virtuous person does not become a consequentialist motivated by maximizing certain outcomes. Rather, the virtuous person is motivated by compassion to minimize harm in a non-ideal situation, and they would appear callous or cruel if they adopted a proscription on direct animal harm knowing that it would create or allow much more animal harm (Abbate, 2014; Allen and Hampton, 2020; Beausoleil, 2020). Just as Hursthouse (2011) explains how a virtuous person who wrings the neck of an injured bird does so from compassion (Table 1), so it is the case that the virtuous conservationist who adopts a lethal conservation policy in a tragic situation does so from compassion (Footnote 1). The Compassionate Conservationist that kills or harms animals is not cold and heartless; they acknowledge the harm and respond appropriately to it even when it is morally right to cause it, and they grieve the loss and tragedy of the situation when appropriate.

Compassionate Conservationists are therefore obliged to acknowledge that a virtue-based approach cannot guarantee the conservationist will not adopt policies that harm or kill animals. Wallach et al. (2018) explain that the virtuous person will “make efforts to not inflict intentional and unwarranted suffering,” which allows the possibility that the virtuous person may inflict intentional but warranted suffering. Wallach et al. (2020b) also think compassion will incline the virtuous person to live by the Golden Rule. Although they recognize that difficult decisions must often be made for which there is no easy answer, they maintain that virtuous compassion would replace the “default of domination” (i.e., Traditional Conservation) with their “default of compassion” (Wallach et al., 2020b). Adopting this default of compassion, however, “does not mean that one never harms a person nor that there cannot be variations in our obligations to different persons” (Wallach et al., 2020b), leading Batavia et al. (2021) to agree that the Compassionate Conservationist who attends to the complexity of a particular situation may indeed judge that *it is appropriate* to kill or harm animals. When this happens, of course, the virtuous person will grieve appropriately (Batavia et al., 2020). The previous examples and discussion therefore illustrate that policies and practices that cause intentional harm or death to animals can also be compatible with virtue-based Compassionate Conservation.

## Compassionate decision-making for all conservationists

The preceding information shows that Compassionate Conservationists from either a deontological or virtue ethics stripe may both adopt intentional lethal or otherwise harm-causing policies and practices. The underlying philosophy and ethic of the Compassionate Conservation movement does not establish the necessity of a complete refrain from the adoption of intentional harm-causing policies and practices, just as the underlying philosophy and ethic of Traditional Conservation does not always permit the adoption of such policies and practices (Table 1). It therefore remains to be seen how Compassionate Conservation philosophy is any different in practice from the more traditional consequentialist-type of conservation philosophy. After all, compassion and empathy toward all animals has always been present in traditional conservation (Fleming, 2018), although perhaps not in such explicit terms, and it is not as though Traditional Conservationists take pleasure in inflicting animal harm and death (Hayward et al., 2019). A Compassionate Conservationist might argue that they adopt a novel decision-making process that generates compassionate (read: non-harmful) conservation policies and practices. But in this section we show that this is not the case, and in practice, their decision-making process often mirrors the decision-making process of Traditional Conservationists (Table 1).

Proponents of Compassionate Conservation claim to provide a distinct form of conservation decision-making, specifically, one that values individual animals when deliberating among potential conservation policies and practices. For example, Ramp and Bekoff (2015) explain that their view “brings empathy into decision-making alongside other values” in a way that may allow for animal interests to possibly supersede human interests. Wallach et al. (2020b) further explains that this position “recognizes that the interests and agency of all sentient beings should be protected in conservation practice,” and Batavia et al. (2021) add that “compassion should animate and inspirit conservation actions, intentions, and interactions.” But there is a significant argumentative gap in these assertions because the proposed type of compassionate decision-making does not require the strict adoption of “do-no-harm” or “peaceful coexistence” policies and practices. As described above, the virtuously compassionate person may well adopt lethal or harm-causing policies or it may be that the miniride or worse-off principles apply in a particular situation (Table 1). To show that compassion for individual animals requires absolute refrain from lethal or harm-causing conservation policies, the Compassionate Conservationist must first show that such refrain is warranted in the many and varied tragic situations commonly experienced by conservationists.

Compassionate Conservationists further advocate for non-lethal or harmless policies on grounds that they promote the best overall outcome (Ramp, 2013; Ramp and Bekoff, 2015; Wallach et al., 2015, 2018; Baker and Winkler, 2020; Bekoff, 2020) or that lethal practices are ineffective (Ramp et al., 2013; Bekoff and Pierce, 2017; van Eeden et al., 2018; Lynn et al., 2019; Cassini, 2020). As examples of this contention, consider the following statements from proponents:

“Although killing for conservation may aim to serve important objectives, it also entails injury, distress, diminished quality of life, and death for wildlife individuals (Dubois et al., 2017). These programs also usually fail to define, defend, and meet clear objectives (Ramp and Bekoff, 2015). A commitment to compassion can allay practices that intentionally and unnecessarily harm wildlife individuals without fundamentally compromising critical conservation goals.” (Wallach et al., 2018)

“Often, it seems as if the only and easiest solution is to kill the “problem animals” and move on to the next situation, in a never ending series of conflicts. However, killing simply does not work in the long run.” (Bekoff, 2015)

“Most lethal programs are not evidence based or even monitored (e.g., Reddiex and Forsyth, 2007; Dubois et al., 2017; Doherty et al., 2019). Many lethal programs are known to fail or exacerbate extinction risk by disrupting social and trophic interactions (e.g., Wanless et al., 2007; Bergstrom et al., 2009; Wallach et al., 2010); curtailing emergent ecological dependencies (Schlaepfer et al., 2011); harming species that now thrive only outside their native ranges (Wallach et al., 2020a,b); and overlooking the underlying human-caused ecological changes shaping species interactions that result in extinctions (Doherty et al., 2019). Additionally, and importantly, the normalization of lethal programs crowds out motivation to invest in research on compassionate alternatives (Dubois et al., 2017).” (Wallach et al., 2020a,b)

“Contrary to Loss and Marra’s (2018) claims that the scientific consensus is consistent with their views that cats are a global threat to biodiversity, the actual scientific consensus is that cats can, in certain contexts, have suppressive population-level effects on some other species (Twardek et al., 2017). This is something that is true of all predators, native or not (Wallach et al., 2010). Thus, cats should not be profiled as a general threat *a priori* and without reference to important factors of ecological context, situational factors, clear definition of harms, and evidence thereof.” (Lynn et al., 2019)

Since the ethical foundation of Compassionate Conservation does not require harmless policies and practices (as described above), and conservationists regularly find themselves in situations in which animals are going to be harmed no matter what they do (Table 1), the Compassionate Conservationist needs to show that harmless policies and practices are the best policies and practices for all individual animals… which begins to sound a lot like the consequentialist approach advocated by Traditional Conservationists. Wallach et al. (2018) offer examples of Compassionate Conservation that are proposed as being more effective than lethal alternatives, although they still implicate the Compassionate Conservationist in some harm. For instance, they praise a program that deployed maremma guardian dogs (*Canis familiaris*) to protect a colony of little penguins (*Eudyptula minor*) from red foxes (*Vulpes vulpes*). And while the penguin population recovered with no red foxes being intentionally killed by people, it is not altogether harmless because this type of practice indeed involves substantial harm to the foxes, guardian dogs, and penguins (see Allen et al., 2019; see also Table 1). Wallach et al. (2015, 2020a,b) also propose cessation of killing dingoes (*Canis familiaris*) for livestock protection on grounds that protecting dingoes would allow them to then kill substantial numbers of other competing predators and thereby convey some assumed net benefits to shared wildlife prey species, not to mention the additional killing of livestock by dingoes (see Allen and Hampton, 2020). How these proposed examples of compassionate conservation practice result in the best overall welfare outcomes for all animals is unclear given the substantial harms to animals caused by these proposals, but they do show two things. First, Compassionate Conservationists clearly support practices that cause intentional animal harm and death to animals, especially the outsourcing of harm to non-human animals; and second, they support these practices on grounds that they are more effective at achieving conservation goals. This reveals that the primary difference in point of view between Compassionate Conservationists and Traditional Conservationists is not so much a question of the underlying ethics, motivations or morality of a given practice, but a more a question of the efficacy of various conservation practices at minimizing animal harm. Compassionate Conservationists and Traditional Conservationists may each end up implementing the same management action in response to many of the common scenarios experienced by conservationists (Table 1).

## Discussion

Compassionate Conservationists advocate for more hands-off conservation policies, including do-nothing policies, more often than Traditional Conservationists do because the latter give considerable weight to respecting the interests of all animals *qua* individuals (Batavia and Nelson, 2017). As Coghlan and Cardilini (2021) explain, Traditional Conservation “readily embraced and still embraces mass killing and poisons and technologies that cause great suffering, often implemented without adequate knowledge of the likely consequences and effectiveness of those actions.” By contrast, the Compassionate Conservationist apparently gives close scrutiny to policies and practices that harm animals, and it just so happens that more often than not, they argue, the evidence shows that a non-harmful policy is the best policy overall. But here is where critics of Compassionate Conservation are apt to point out that it is rarely the case that there is a policy that involves no animal harm, intentional or otherwise; and indeed, there is strong evidence that adopting a policy that involves some direct animal harm actually minimizes overall animal harm (Fleming et al., 2012; Russell et al., 2016; Hampton et al., 2019; Hayward et al., 2019; Allen and Hampton, 2020; Callen et al., 2020; Griffin et al., 2020). For example, consider Boks (2018) presentation of the back-and-forth between Wallach and Fleming discussing the management of European wild rabbits (*Oryctolagus cuniculus*) in Australia:

Wallach: “Killing rabbits, and other unwanted species, has not provided the cure to Australia’s environmental problems, because ultimately these problems are of our own making. Persecution of Australia’s apex predator, the dingo, alongside other human activities such as overgrazing with livestock, have been highly detrimental. Rabbits are merely the scapegoat. Dingoes, particularly when socially stable, can limit rabbit densities. Providing space for dingoes to assume their ecological function offers a way forward that is both effective and ethical.”

Fleming: “This is untrue on many levels. For a start, killing many rabbits has been shown to be the only effective way of limiting their ecological and agricultural damage (McLeod and Saunders, 2014). The release of myxoma virus in the 1950s and rabbit hemorrhagic disease in the 1990s and last year, both of which killed millions of rabbits, and ongoing poisoning, ripping and shooting have resulted in 20 years of vegetation restoration and saved endangered mammals (Pedler et al., 2016). The reason for this is simple. Peter Bird, Greg Mutze and colleagues in South Australia have shown that rabbits in extremely low densities (equivalent to less than 1 rabbit in an area the size of Adelaide Oval) prevent recruitment of some species including coastal she-oak, which is the structural backbone of the fragile, coastal ecosystem in which they occur.”

The above exchange illustrates that this disagreement is not about the welfare, ethics or morality of killing rabbits or dingoes, for recall that it may well be acceptable depending on the situation (Table 1). Instead, the focus of the disagreement is on the effectiveness of conservation efforts that kill rabbits, with Wallach describing them as ineffective and Fleming describing them as effective. This is because all conservation options in this situation involve animal harm; and so the challenge from a compassionate standpoint, as evidenced in the Wallach passage, is to identify the best practical means of achieving the least amount of harm. If it could be shown that killing some rabbits is the most effective way to achieve the best possible outcome for all individuals involved, then Wallach and other Compassionate Conservationists ought to be open to adopting it. Similarly, if it could be shown that enabling dingoes to kill rabbits is the most effective way to achieve the best possible outcome in the situation, then Fleming and other Traditional Conservationists ought to be open to adopting it.

This all leads to the observation that Compassionate Conservationists typically advocate for similar decision-making processes as Traditional Conservationists in practice, though each may not realize it or like to admit it. Compassionate people care about individual animals and respond appropriately to harm. In a situation in which no harmless options are available, compassionate people will carefully scrutinize and weigh the associated costs of inaction (e.g., how many animals will be harmed by a hands-off approach) to action (e.g., how many animals will be harmed by approaches A, B, and C etc.). Their compassion toward animals will then motivate them to select the course of action that minimizes animal harm, either through the virtue of compassion itself or *via* the miniride or worse-off principles espoused by the deontologist. If inaction will lead to significantly greater harm to greater numbers of animals, say through overpopulation or hyperpredation, then compassionate people will adopt a course of action that invariably involves them in some form of animal harm. Although motivated principally by compassion for individual animals, this decision-making process closely mirrors the decision-making process of dispassionate consequentialists and arrives at the same practical endpoint (Table 1). Internally, of course, there is a profound difference, for the deontologist simply wants to do their moral duty, the virtuous person simply wants to express the most compassion, and the consequentialist simply wants to minimize harm. Externally, however, the actions of a Compassionate Conservationist can be somewhat indistinguishable from a Traditional Conservationist, and vice versa.

There are some limitations to this view, however, especially for our characterization of the virtue-based compassionate conservationist. Our discussion focused on the similarities shown by different types of conservationist in their own words as they attempt to resolve the practical problems they face on a daily basis, but the virtue-based compassionate conservationist may be motivated by other considerations or may adopt a different account of virtue ethics than that which Compassionate Conservationists appeal to (Akrivou et al., 2019, 2020). For example, a Compassionate Conservationist may argue that practical wisdom suggests that intentionally harming animals may undermine the moral character of the conservationists themselves, thereby threatening their flourishing, and may undermine their ability to deal with conservation challenges in the future. Although we are skeptical that different kinds of virtue ethics would support an apodictic refrain on intentional animal harm, this would be a fruitful line of inquiry. Moreover, as their experience and capability to deal with conservation problems grows, the maturation of their character may lead to new ways of displaying virtue, which may be different to the way we have described here. What was virtuous before may not be considered virtuous now, and what is virtuous now may not be considered virtuous in the future. Along with deontological compassionate conservationists and consequentialist traditional conservationists, the course of action taken by each type of conservationist (Table 1) may change as new information or better evidence is forthcoming.

## Conclusion

We have shown that: (1) Compassionate Conservation as espoused by its proponents (inclusive of deontological-based and virtue-based) and Traditional Conservation approaches can follow similar decision-making processes that lead both to arrive at similar practical outcomes for animals; that (2) traditional forms of conservation which involve direct harm and killing of animals are consistent with the ethical philosophies underpinning Compassionate Conservation; and that (3) the perceived disagreement between the two approaches is not really about the underlying ethics, motivations or morality of the different views, but is instead more about the efficacy of various animal management practices at reducing harm. Thus, an important issue for continuing research is to examine whether or not conservation policies and practices are effective at minimizing animal harm, and if not, what policies and practices are. Since we live in a non-ideal world where there is usually no harm-free option available to conservationists, we suspect that compassion may motivate the adoption of traditional policies and practices that cause intentional animal harm more often than Compassionate Conservationists would like to admit. We also suspect that debates about the utility of Compassionate Conservation will converge on issues surrounding the actual outcomes for fauna, including the effectiveness of various policy and practice recommendations.

## Author contributions

All authors listed have made a substantial, direct, and intellectual contribution to the work, and approved it for publication.

## Conflict of interest

The authors declare that the research was conducted in the absence of any commercial or financial relationships that could be construed as a potential conflict of interest.

## Publisher’s note

All claims expressed in this article are solely those of the authors and do not necessarily represent those of their affiliated organizations, or those of the publisher, the editors and the reviewers. Any product that may be evaluated in this article, or claim that may be made by its manufacturer, is not guaranteed or endorsed by the publisher.

## Footnotes

^1^Bobier, C., Allen, B. L. (2022). The virtue of compassion in compassionate conservation. *Conserv. Biol.* 36, e13776: doi: 10.1111/cobi.13776.
